# A novel oral micellar fenretinide formulation with enhanced bioavailability and antitumour activity against multiple tumours from cancer stem cells

**DOI:** 10.1186/s13046-019-1383-9

**Published:** 2019-08-22

**Authors:** Isabella Orienti, Valentina Salvati, Giovanni Sette, Massimo Zucchetti, Lucilla Bongiorno-Borbone, Angelo Peschiaroli, Lello Zolla, Federica Francescangeli, Mariella Ferrari, Cristina Matteo, Ezia Bello, Antonio Di Virgilio, Mario Falchi, Maria Laura De Angelis, Marta Baiocchi, Gerry Melino, Ruggero De Maria, Ann Zeuner, Adriana Eramo

**Affiliations:** 1 Department of Pharmacy and Biotechnology, University of Bologna, Bologna, Italy; 2 Department of Oncology and Molecular Medicine, Istituto Superiore di Sanità, Rome, Italy; 3 Istituto di Patologia Generale, Università Cattolica del Sacro Cuore, Rome, Italy; 4 Department of Oncology, Istituto di Ricerche Farmacologiche Mario Negri IRCCS, Milan, Italy; 5 Department of Experimental Medicine, TOR, University of Rome “Tor Vergata”, Rome, Italy; 6 National Research Council of Italy (CNR), Institute of Translational Pharmacology IFT, Rome, Italy; 7 Department of Ecological and Biological Sciences, University of Tuscia, Viterbo, Italy; 8 Service for Biotechnology and Animal Welfare, Istituto Superiore di Sanità, Rome, Italy; 9 National AIDS Center, Istituto Superiore di Sanità, Rome, Italy; 10 Fondazione Policlinico Universitario “A. Gemelli” - I.R.C.C.S, Rome, Italy

**Keywords:** Fenretinide, Bioavailability, Solubility, Antitumour activity, Cancer stem cells, Solid tumours, Drug delivery, Pharmacokinetics, Cancer therapy

## Abstract

**Background:**

An increasing number of anticancer agents has been proposed in recent years with the attempt to overcome treatment-resistant cancer cells and particularly cancer stem cells (CSC), the major culprits for tumour resistance and recurrence. However, a huge obstacle to treatment success is the ineffective delivery of drugs within the tumour environment due to limited solubility, short circulation time or inconsistent stability of compounds that, together with concomitant dose-limiting systemic toxicity, contribute to hamper the achievement of therapeutic drug concentrations. The synthetic retinoid Fenretinide (4-hydroxy (phenyl)retinamide; 4-HPR) formerly emerged as a promising anticancer agent based on pre-clinical and clinical studies. However, a major limitation of fenretinide is traditionally represented by its poor aqueous solubility/bioavailability due to its hydrophobic nature, that undermined the clinical success of previous clinical trials.

**Methods:**

Here, we developed a novel nano-micellar fenretinide formulation called bionanofenretinide (Bio-nFeR), based on drug encapsulation in an ion-pair stabilized lipid matrix, with the aim to raise fenretinide bioavailability and antitumour efficacy.

**Results:**

Bio-nFeR displayed marked antitumour activity against lung, colon and melanoma CSC both in vitro and in tumour xenografts, in absence of mice toxicity. Bio-nFeR is suitable for oral administration, reaching therapeutic concentrations within tumours and an unprecedented therapeutic activity in vivo as single agent.

**Conclusion:**

Altogether, our results indicate Bio-nFeR as a novel anticancer agent with low toxicity and high activity against tumourigenic cells, potentially useful for the treatment of solid tumours of multiple origin.

**Electronic supplementary material:**

The online version of this article (10.1186/s13046-019-1383-9) contains supplementary material, which is available to authorized users.

## Background

Most tumours are heterogeneous and contain a small population of CSC that are believed to play a crucial role in tumour progression, drug resistance, recurrence and metastasis in multiple malignancies [30]. Thus, several potential approaches to kill therapeutically-resistant CSC have been deeply explored and, besides overcoming primary intrinsic resistance of tumour cells, they have been proved effective against tumour relapse and metastasis, as well [12, 22]. However, in view of the plasticity of tumour cells, drugs with broad toxicity against different tumour cell populations would to be preferred to achieve long-term antitumour efficacy [40]. Another huge obstacle to treatment efficacy is the inefficient drug absorption into the tumour environment mainly due to low systemic availability which limits the drug distribution to the different body compartments, including the tumor. Moreover, the unspecific toxicity of the antitumor drugs strongly restrains dose-escalation as a mean to improve the systemic availability [45]. Properly designed nanoformulations may offer the possibility of increasing the drug systemic availability, thus improving the drug concentration at the tumour site to levels suitable at eliciting an antitumour response.

In nanoformulations the drug is generally enclosed in nanostructured materials (nanoparticles) providing drug aqueous solubilization at extents depending on the overall characteristic of the system. Based on the physico-chemical properties of the materials the nanoparticles may be nanomicelles, nanocapsules, nanospheres etc. [23]. They are mainly administered by oral or intravenous route. In intravenous administration the nanoparticle role changes with the tumor characteristics. In tumors with vascular discontinuities the nanoparticle function is to transport the drug in circulation avoiding release until extravasation takes place through the discontinuities of the tumor capillaries where the nanoparticle accumulation provides increased drug concentration in the tumor environment with respect to the other body compartments [18, 25, 34].

In oral administration the nanoparticle role is to increase the drug solubilization and absorption in the gastrointestinal environment to enhance drug plasma concentration and biodistribution at suitable levels for drug activity. The nanoparticles do not cross the gastrointestinal wall and lipophilic drugs such as fenretinide are primarily absorbed by the lipid absorption pathway mediated by chylomicrons (Barry J. [26]).

Nanoparticles for oral administratioin have been extensively studied to improve the therapeutic efficacy of antitumor drugs [11]. Oral lipid nanoparticles, in particular have been proved to increase solubility, chemical stability, epithelium permeability and bioavailability of hydrophobic bioactive compounds [17].

Moreover, lipid nanocarriers offer new possibilities of obtaining therapeutic drug levels by oral administration, the most simple and reliable administration route. Indeed they have been proved to increase solubility, chemical stability, epithelium permeability and bioavailability of hydrophobic bioactive compounds [17].

Fenretinide (4-HPR) is a synthetic retinoid which emerged as a promising anticancer agent based on numerous in vitro and animal studies as well as chemoprevention clinical trials (B. J. [29, 48, 49]). It is reported to induce cytotoxicity by multiple mechanisms (Holliday, Cox, Kang, & [27]; B. J. [28, 35, 38, 51, 52]). Significantly, fenretinide has shown the potential to target CSC-associated molecular targets, further increasing its therapeutic value [1, 10, 33, 53]. An oral formulation of fenretinide, consisting of soft gelatin capsules containing fenretinide in corn oil and polysorbate, is currently available at the National Cancer Institute (NCI-FeR). However, despite promising in vitro cytotoxicity, its antitumour activity in clinical trials (both as a cancer therapeutic and chemopreventive agent) remains limited, likely due to poor bioavailability of this drug formulation requiring the administration of excessively elevated drug doses that constrain drug tolerability and patient compliance [3, 4, 15]. Numerous attempts have been made to develop fenretinide formulations capable of increasing the drug aqueous solubility and bioavailability to levels that can elicit a satisfactory therapeutic response. Promising, although still unsatisfactory results have been obtained in a trial with neuroblastoma children, while in adult patients (lymphoma) the high drug doses required to achieve therapeutic plasma drug concentrations strongly limited patient compliance causing gastrointestinal toxicity and (reversible) nyctalopia. Improved efficacy of oral fenretinide, when co-administered with other drugs, has been reported, possibly due to lower drug concentration required for synergistic activity or to increased fenretinide exposure induced by co-administered drugs [4, 8, 20, 27, 37]. Intravenously infused fenretinide as a soy oil-in-water emulsion showed promising results in patients with hematologic malignancies [32]. Lastly, all these studies suggested that improved fenretinide formulations are needed to couple acceptable drug doses, tolerability and patient compliance with significant systemic exposure, aiming at satisfactory therapeutic efficacy also as single agent treatment for both adult and pediatric patients.

We recently reported a new fenretinide-cyclodextrin complex, which has a higher solubility and bioavailability as compared to previous formulations [36]. However such complex requires intravenous administration, which limit the potential clinical use of this drug particularly for extended treatment periods. Now, we developed a nano-formulation of fenretinide, called bionanofenretinide (Bio-nFeR) with the aim to combine a high bioavailability and low toxicity with the suitability for oral administration.

We developed a new nano-formulation of fenretinide, called bionanofenretinide (Bio-nFeR) designed with the aim to raise the poor oral fenretinide bioavailability, that has prevented its clinical use in spite of its considerable antitumour activity and low toxicity profile, until now. Bio-nFeR is based on ion pair formation between fenretinide and phosphatidylcholine triggering the spontaneous formation of micelles, in water, containing fenretinide in their inner core.

Bio-nFeR displayed very promising antitumour activity, superior than NCI-FeR, against CSC of various origin in vitro at low concentrations, and in CSC-derived tumour xenografts of lung, colon and melanoma origin, reaching higher drug concentration in blood and therapeutic drug tumour levels within xenografts, in absence of toxicity.

## Materials and methods

### Chemicals

4-HPR (fenretinide) was purchased from Olon Spa (Milan Italy). L-α-phosphatidylcholine from egg yolk, glyceryl tributyrate and all the other chemicals were purchased from Sigma-Aldrich. Deuterated fenretinide (C_26_H_29_D_4_NO_2)_ used as internal standard (IS), was obtained from Tocris Bioscience (Bristol, UK).

### Bio-nFeR preparation

Fenretinide was homogeneously mixed with L-α-phosphatidylcholine and glyceryl tributyrate dispersed in alkaline ethanol to a final weight ratio 1:9:1 w:w:w respectively. Ethanol was removed from the mixture in a rotary evaporator and the dry residue stored at − 18 °C until use. Reconstitution of the dry residue to Bio-nFeR nanomicelles was accomplished by dissolving the residue in water at 30 °C in an ultrasound bath where a wave frequency of 40 kHz was applied for 1 h to the dissolving phase. The dissolved residue was subsequently filtered through 0.4 um pore filters to obtain homogeneously dispersed nanomicelles of controlled mean size. To compare Bio-nFeR with the soft gelatin capsules representing the standard formulation most used in clinical trials for fenretinide, we reconstituted the capsules content according to the directions provided by National Cancer Institute per capsule (fenretinide: 100 mg, corn oil: 704 mg, polysorbate 80: 60 mg). The final concentration in the reconstituted content of the NCI capsules (NCI-FeR) being 11.6% w:w fenretinide:internal capsule components, as per the NCI instructions.

### Solubilization ability, encapsulation efficiency and drug loading capacity of bio-nFeR towards fenretinide

To evaluate the solubilization ability of Bio-nFeR towards fenretinide, reconstitution of the dry residue to nanomicelles was carried out at concentrations increasing from 50 to 300 mg/ml, corresponding to the concentration range used in this study. After filtration through 0.4 um pore filters, the nanomicelles were diluted (1:3, v:v) with an ethanol:water (1:1, v:v) mixture and spectrophotometrically analysed at 364 nm for their fenretinide content. As a comparison the same evaluation was carried out on empty nanomicelles prepared by the same procedure. The encapsulation efficiency was obtained as the ratio between the drug content in the nanomicelles and the drug loaded in the dry residue used for the nanomicelles preparation. The drug loading capacity was calculated as the drug weight content in the nanomicelles per unit weight of nanomicelles, obtained by the weight of the freeze-dried nanomicelle suspensions.

### Light scattering characterization of bio-nFeR

Particle size, polydispersity and zeta potential were measured at 37 °C on nanomicelle dispersions obtained by reconstitution of the dry residue at 50 mg/ml and diluted 1:100 with water (Malvern Instruments, Worcestershire, UK). A minimum of 12 measurements per sample were made. Results were the combination of three 10-min runs for a total accumulation correlation function time of 30 min. The results were volume-weighted.

### Feneretinide release from bio-nFeR

The release of fenretinide from Bio-nFeR was measured in three different media: HCl solution (pH 1.2), phosphate buffer solution (pH 6.8) and phosphate buffer solution (pH 6.8) containing sodium taurocholate (3.0 mM), to simulate the main physico-chemical characteristics of the gastrointestinal fluids [19]. The nanomicelles obtained by reconstitution of the dry residue at 50 mg/ml were diluted (1:10) with the different media and placed (1 ml) in a dialysis bag (Mw cutoff 5KD) which was subsequently sealed and immersed in a receiving compartment containing water (20 ml) and n-octanol (5 ml). N-octanol was added as a drug-extraction phase simulating the presence of cell membranes in vivo [31]. The system was thermostated at 37 °C. At increasing time intervals spectrophotometric analysis in the releasing compartment was carried out as described above.

#### Confocal microscopy

Images were taken by a FV1000 confocal microscope (Olympus, Tokyo, Japan), using a (Olympus) plan apo objective 60× oil A.N. 1,42. Excitation light for fenretinide was obtained by an Argon Ion Laser (488 nm) and emission was recorded from 495 to 550 nm. Images were taken also by a Nikon C1s confocal laser scanning microscope, equipped with a Nikon PlanApo 60, 1.4-NA oil immersion lens.

### Cell culture, drug treatments and cell viability assay

Patient-derived CSC lines were obtained from primary, melanoma, glioblastoma, lung and colon cancers and cultured as previously described [9, 14, 42–44].

For drug treatments and cell viability assays, 3000 cells were plated in triplicate in 96-well plates and left untreated or exposed for 72 h to Bio-nFeR (stock solution 200 mg/ml freshly dissolved in water) or NCI-FeR (20 mg/ml dissolved in corn oil and polisorbate 80). Cell viability was detected with Cell TiterGlo (Promega).

### Pharmacokinetic studies

#### Animals

The experiments were performed in CD1 female mice, 7 weeks of age, (body weight 25 ± 2 g) obtained from Envigo RMS SrL (Udine, Italy). They were maintained under specific-pathogen-free conditions with constant temperature and humidity, according to institutional guidelines regularly checked by a certified veterinarian. Animal experimentation was conducted in conformance with the following laws, regulations, and policies governing the care and use of laboratory animals: Italian Governing Law (D. l. 26/2014; Authorization n.19/2008-A issued March 6, 2008 by the Ministry of Health). Mario Negri Institutional Regulations and Policies providing internal authorizations for persons conducting animal experiments (Quality Management System Certificate –UNI EN ISO 9001:2008 – Reg. N 86121). The experimental protocol was reviewed by the Mario Negri Institute Ethical Committee and approved by the Italian Ministry of Health (Aut. Min. No 489/2016-Pr).

#### Drug and formulations

The pharmacokinetics of oral Bio-nFeR was investigated in comparison to that of the reference formulation NCI-FeR, after single administration of multiple doses and after chronic administrations, daily for 2 weeks or bi-daily for 1 week. Bio-nFeR was reconstituted at 20 mg/mL fenretinide to allow the administration of 200 mg/Kg fenretinide that corresponded to the highest dose. The doses of 100, 50 and 10 mg/Kg were administered by using serial dilution of the 20 mg/mL solution.

NCI-FeR prepared, as per the NCI instructions, at 11.6% w:w fenretinide:internal capsule components, was diluted with corn oil to obtain the treatment solutions at the concentrations of 20, 15 and 10 mg/mL, corresponding to the administered doses of 200, 150 and 100 mg/Kg respectively.

#### Treatments and samples collection. Acute study

The oral pharmacokinetics of Bio-nFeR were investigated in four independent groups of 24 mice, randomized to receive different treatment with the following fenretinide equivalent doses: 10, 50, 100 and 200 mg/Kg. The drug was administered by oral gavage and after treatment, blood samples were taken at 15 and 30 min and at 1, 2, 4, 8/10, 24 and 48 h and collected in heparinized tubes from the retro-orbital plexus of the mice under isoflurane anesthesia. Mice were then sacrificed by cervical dislocation and liver, kidney and brain were removed and immediately frozen in dry-ice for future determination. To obtain plasma, blood was centrifuged at 4000 rpm for 10 min at 4 °C. To evaluate fenretinide and metabolites excretion, urine and faeces were collected over a 0–24 h and 24–48 h periods. The collection was performed in three mice per group of treatment, individually housed into metabolic cages for urine and faeces collection. All the biological samples were stored at − 20 °C until analysis. To compare the absorption of Bio-nFeR to NCI-FeR and to study the bioavailability, another independent groups of 24 mice were randomized to receive an oral dose of 200 mg/Kg of NCI-FeR. Blood samples were taken at the time reported above. Twenty-four hours fractions of urine and faeces were collected in three mice per group of treatment. Plasma and the other biological specimens were stored at − 20 °C until analysis. Chronic study: the oral pharmacokinetic of Bio-nFeR in comparison with NCI-FeR were also investigated after chronic administrations. Mice were divided in three independent groups, randomized to receive a single treatment of 150 mg/Kg or different repeated treatments of 150 mg/Kg, daily for 2 weeks (12 days on and 2 days off), or 100 mg/Kg bi-daily for 1 week (5 days on 2 days off). Bio-nFeR and NCI-FeR were administered by oral gavage, collecting blood samples at 0.5, 1, 2, 4, 8/10 and 24 h, the day of the single treatment and at 0, 1, 2, 4, 8/10, 24 and 48 h after the last dose of both the chronic treatments. The procedures of blood collection, plasma preparation, sacrifice of mice and storing of samples were as reported in acute study. Also during chronic treatment, excretions of fenretinide and metabolites in urine and faeces were determined collecting the specimens in three mice per group, both at the start of treatment and at the end of the repeated administration. All the collected samples were stored at − 20 °C until analysis.

#### Drug assay and pharmacokinetic elaboration

The determination of fenretinide in plasma of mice and the others biological samples was determined according to a method specifically developed and validated for the quantification of fenretinide (4-HPR) based on liquid chromatography-tandem mass spectrometry (LC–MS/MS) (*manuscript in preparation*). The procedure of sample preparation and quantification can be summarized as follows. 30 μL aliquot of plasma was added with 3 ng of deuterated internal standard (^2^H_4_ 4-HPR), deproteinized by mean of 90 μL of acetonitrile and centrifuged 5 min at 13200 rpm at room temperature. The supernatant was recovered and 5 μL injected into a HPLC system (Series 200, Perkin Elmer, MA, USA). Chromatographic separation was achieved on a Gemini-C18 column (50 mm × 2.0 mm, 5 μm particle size; Phenomenex Inc., Torrance, CA, USA) at 32 °C protected with a Security Guard™ ULTRA cartridges C18. The detection obtained by Mass spectrometric detection was carried out on a triple quadrupole API 4000 mass spectrometer (Sciex, MA, USA) equipped with atmospheric pressure chemical ionization source (APCI) operating in positive ion mode monitoring the transitions 392.3 > 283.2 m/z for 4-HPR and 396.3 > 283.2 m/z for the deuterated internal standard. To assay study samples, a calibration curve of six points (range: 1.0–500.00 ng/mL) was prepared in control mouse plasma assessing the precision and the accuracy of the run analyzing at the same time a set of freshly prepared quality controls samples. The Limit Of Quantification of the method was 1 ng/mL in plasma and urine and 50 ng/g in faeces.

### Xenografts generation, antitumour activity studies and tumour pharmacokinetics of bio-nFeR

Cell suspensions of lung and colon cancer and melanoma CSC lines were mixed 1:1 with Growth Factor-Reduced Matrigel (Beckton Dickinsons) and injected subcutaneously in both flanks of 5-week-old female NOD-SCID (non-obese diabetic/severe combined immunodeficiency) mice in the case of colon and melanoma or NSG (non-obese diabetic/severe combined immunodeficiency gamma chain deficient) mice in the case of lung () (Charles River, Wilmington, MA, USA). For drug treatment, when tumours reached a mean diameter of 0.5 cm, groups of 6 mice were randomized into 3 groups and treated with vehicle, with Bio-nFeR (50,100 or 150 mg/kg as described) freshly dissolved in sterile water or with the standard formulation NCI-FeR that reproduce NCI capsule (50 or 100 mg/Kg). Treatments were administered by oral gavage 5 days on and 2 days off for 3 weeks. Tumours were measured twice a week using a caliper, and mice were monitored for signs of drug-induced toxicity and weighed regularly. At the end of treatments, in a study paralleling the assessment of antitumour activity, tumours and blood samples were collected for pharmacokinetic and molecular studies 3 hours after the last drug administration. Collection of blood, plasma separation and storing were as previously described. Immediately after blood collection mice were sacrificed by cervical dislocation, tumours removed rapidly frozen in liquid nitrogen and stored in small pieces at − 80 °C for subsequent fenretinide determination, protein and lipid analyses. All procedures were performed with the approval of the Ethics Committee for Animal Experimentation.

### Lipid analyses: HPLC-HRMS

Extracted tissue samples (20 μl) were injected into an Ultra High Performance Liquid Chromatography (UHPLC) system (Ultimate 3000, Thermo). Samples were loaded onto a Reprosil C18 column (2.0 mm × 150 mm, 2.5 μm- Dr. Maisch, Germany) for lipids separation. Chromatographic separations were achieved at a column temperature of 30 °C; and flow rate of 0.2 mL/min. For lipids multi-step gradient program was used. It started with 8% solvent A (ddH20, 20 mmol L-1 ammonium formiate; pH 5) to 6% solvent A for 3 min than to 2% solvent A for 35 min and finally to 100% solvent B (methanol) in 30 min. At the end of gradient, the column was reconditioned with 8% solvent A for 5 min.

The UHPLC system was coupled online with a mass spectrometer Q- Exactive (Thermo). The instrument was used with full scan (FS) and a subsequent data dependent acquisition (DDA) mode. The settings for Full scan were as follows: resolution, 70,000; automatic gain control (AGC) target, 3e6; maximum injection time (IT), 100 ms; and scan range, m/z 150–2000. The remaining settings for DDA mode were as follows: resolution, 17,500; AGC target, 1e5; maximum IT, 50 ms; isolation window, m/z1.0, HCD with stepped normalized collision energy (NCE), 30. Calibration was performed before each analysis against positive or negative ion mode calibration mixes (Piercenet, Thermo Fisher, Rockford, IL) to ensure sub ppm error of the intact mass. Lipid identification was performed with LipidSearch software (Thermo) that processes LC-MS data, to make provide accurate lipid identification.

### Immunofluorescence and confocal analysis of xenografts

Tumour tissue samples were collected in oct, frozen on dry ice and stored at − 80 °C until further use. Six micron tissue sections were cut with cryostat and mounted on coverslips. Tissue sections were fixed with 2% paraformaldehyde, permeabilized with 0.1% Triton X-100/PBS, quenched with 1 M glycine in PBS and incubated with antibodies to KI-67 (Dako, clone MIB-1) followed by secondary anti-mouse antibody coupled with Alexa 488 (Invitrogen). TUNEL assay was performed using in situ cell death detection KIT fluorescein (Roche) following the manufacturer’s instructions. Nuclei were counterstained with DAPI (Invitrogen) and slides were permanently mounted. Images were taken by a FV1000 confocal microscope (Olympus, Tokyo, Japan), using a (Olympus) objective 10×. Excitation light was obtained by a Laser Dapi 408 nm for DAPI, an Argon Ion Laser (488 nm) for Alexa 488. DAPI emission was recorded from 415 to 485 nm, FITC emission was recorded from 495 to 550 nm.

#### Flow cytometry and aldefluor assay

For flow cytometry, 1 × 10^5^ cells were incubated with the APC-conjugated monoclonal mouse IgG1 antibody anti CD44V6 Clone # 2F10 R&D System or negative control APC-conjugated mouse IgG1 (Ebioscience). Alternatively, the equivalent PE-conjugated antibodies were used. Stained cells were analyzed with a FACScanto flow cytometer (Becton Dickinson). The stem cell population expressing ALDH enzymatic activity was detected by means of the Aldefluor™kit (StemCell Technologies, Vancouver, BC, Canada), according to the manufacturer’s instructions. Briefly, 1 × 10^5^ cells were incubated in Aldefluor assay buffer containing ALDH-substrate for45 min at 37 °C; negative control cells were stained using identical conditions in the presence of ALDH inhibitor diethylaminobenzaldehyde (DEAB). Samples were analyzed by FACScanto flow cytometer (Becton Dickinson) and the fluorescence profiles were compared.

#### Immunoblot analyses

For immunoblotting studies, 20 μg of proteins from each sample were resolved on 4–12% polyacrylamide gel electrophoresis NuPAGE Bis-Tris (Invitrogen, Carlsbad, CA, USA) and transferred to nitrocellulose membranes. The following primary antibodies were used: CD44v6 (VFF-18) mouse monoclonal (eBioscience™), c-Kit (D13A2) rabbit polyclonal (Cell Signaling), ALDH1 (clone44/ALDH) mouse monoclonal (Beckton Dikinsons), BMI1 (D20B7) rabbit polyclonal (Cell Signaling), SOX2 (D6D9) rabbit polyclonal (Cell Signaling) and β-actin monoclonal (Sigma-Aldrich) antibodies. Peroxidase-conjugated secondary antibodies were purchased from Amersham™.

### Statistical analysis

Results are expressed as means ± s.d. from an appropriate number of experiments. Differences were analyzed by ANOVA test using GraphPad Prism v.4.0 for Windows (GraphPad Software, San Diego, CA, USA, www.graphpad.com) and the threshold for statistical significance was set at 0.05. *P*-values are displayed on the graphs using a single asterisk for significances ranging from 0.05 to 0.01, two asterisks for values between 0.01 and 0.001 and three asterisks for values below 0.001.

## Results

### Bio-nFeR nanomicelles

**Fig. 1:**
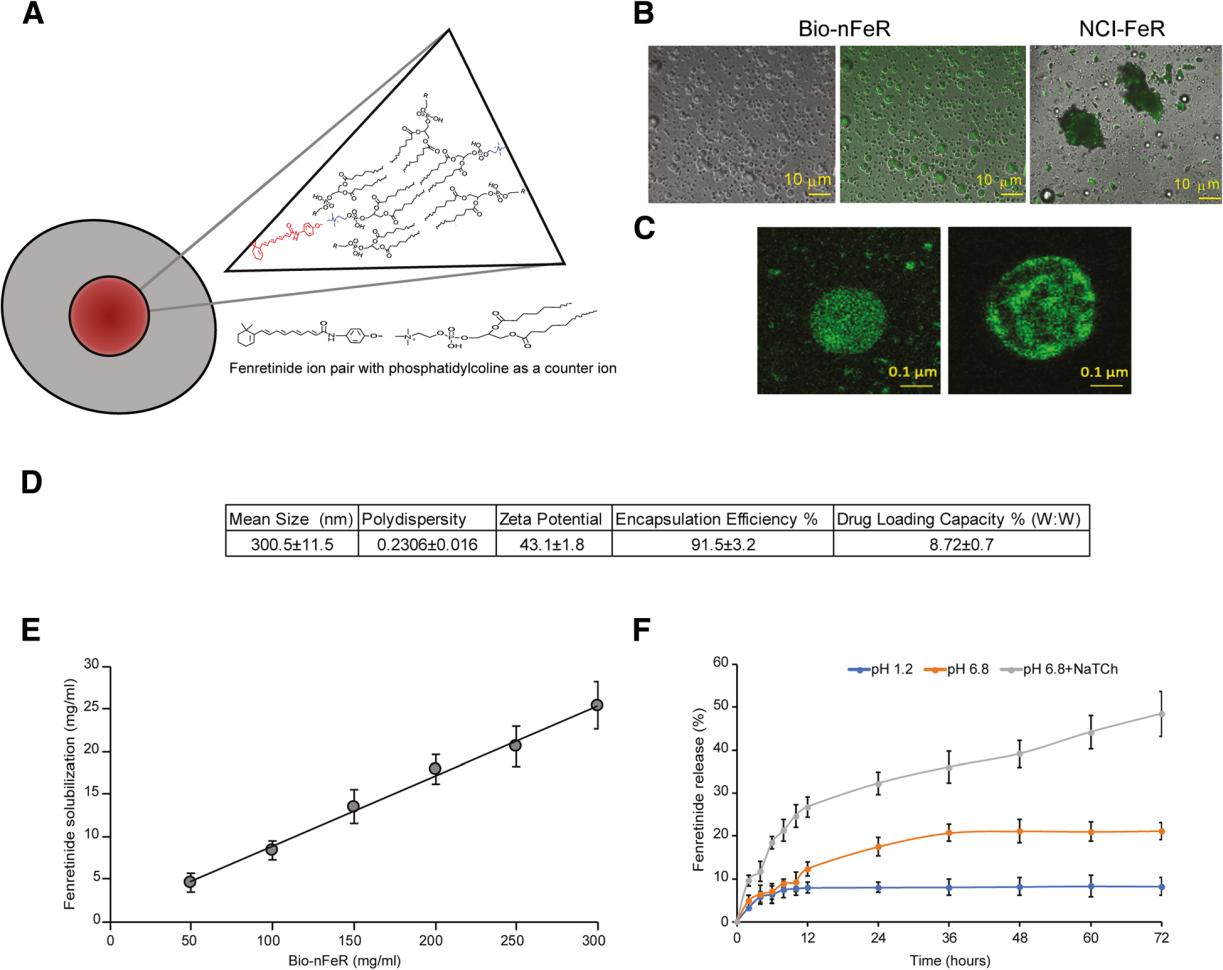
Illustration of the Bio-nFeR biochemical structure and fluorescence microscopic image of drug-containing micelles. **a** Schematic representation of micelles composed of phospholipids in the outer layer and fenretinide in the core. **b** Aqueous dispersions of Bio-nFeR and NCI-FeR formulations. (Left) Optical microscopy image of Bio-nFeR micelles in water. (Middle) Confocal image of Bio-nFeR (the same as in the left) showing the presence of autofluorescent fenretinide within micelles. (Right) Confocal image of NCI-FeR in water forming large aggregates of insoluble material (Images magnification is 60× and 10 μm scale bar is reported). **c** Confocal laser scanning images of Bio-nFeR micelles obtained by fenretinide ionization (left) and micelles obtained by the same procedure but without fenretinide ionization (Images magnification is 60× and 0.1 μm scale bar is reported). **d** Physico-chemical features of Bio-nFeR micelles. **e** Solubilization ability of Bio-nFeR towards fenretinide **f**) In vitro release of fenretinide from Bio-nFeR micelles in HCl solution (pH 1.2), phosphate buffer solution (pH 6.8) and phosphate buffer solution (pH 6.8) containing sodium taurocholate (3.0 mM)

To increase fenretinide bioavailability a new nanomicellar system was prepared by dissolving phosphatidylcholine and glyceryl tributyrate in alkaline ethanol in the presence of the drug. The alkaline solvent, providing ionization of the fenretinide phenolic hydroxyl allowed formation of ion pairs with the choline moiety of the phosphatidylcholine as a counterion. Subsequent evaporation of ethanol provided a semisolid residue containing the fenretinide - phosphatidylcholine ion pairs dispersed in the phospholipid excess-glyceryl tributyrate mixture (Fig. 1a). The dissolution in water of the evaporated residue provided spherical micelles as shown in Fig. 1 B reporting optical microscopy images obtained by dissolution of 100 mg/ml of the evaporated residue before filtration. Indeed, the thermodynamic stability of the fenretinide-phosphatidylcholine ion pair is the driving force for the formation of micelles characterized by a hydrophobic inner core containing fenretinide and an hydrophilic shell formed by the spontaneous assembling of the phospholipid molecules in water where the ion bridge stabilizes the core-shell interface [24].

As a comparison the same preparative procedure was followed by using pure ethanol instead of alkaline ethanol to prevent fenretinide ionization and ion pair formation. Fluorescence microscopy images, obtained by exploiting the spontaneous auto-fluorescence of fenretinide, showed spherical drug localizations inside the micelles prepared by fenretinide ionization, indicating the drug presence in the micellar inner cores in accordance with fenretinide-phosphatidylcholine ion pair formation. Without ionization, on the contrary, concentric drug localizations were obtained, suggestive of liposome-like structures with drug entrapment in the lipophilic phospholipid bilayers (Fig. 1c). The NCI-FeR images showed coarse aggregates related to lack of drug solubilization (Fig. 1b). Dynamic light scattering measures of Bio-nFeR indicated mean sizes in the nanometric scale, good dimensional polydispersity and a negative zeta potential (Fig. 1d). The solubilization ability of Bio-nFeR towards fenretinide, evaluated as the increase in drug concentration with the increase in nanomicelle concentration, was characterized by a linear trend indicative of a micellar-mode of drug solubilization (Fig. 1e). In micellar solubilization the linear trend persists until the micelle concentration become so high to cause self-aggregation and destabilization. In our study the linear trend lasted up to the maximum nanomicelle concentration evaluated (300 mg/ml) providing 25.41 mg/ml fenretinide solubilization, much higher than the aqueous solubility of the pure drug (1.71 10^− 3^ mg/ml).

The release of fenretinide from the nanomicelles was very low either at pH 1.2 and pH 6.8 being 8.2 and 21.19% respectively after 72 h. In the presence of sodium taurocholate, on the contrary, the release was significantly improved being 48.45% at 72 h (Fig. 1f). This indicated stability of the Bio-nFeR nanomicelles towards drug leakage at the different pH of the gastrointestinal environment and their ability to promote intestinal lymphatic transport of fenretinide by the bile activated pathway of lipid absorption [5].

### Pharmacokinetic studies of bio-nFeR after acute and repeated administration

**Fig. 2:**
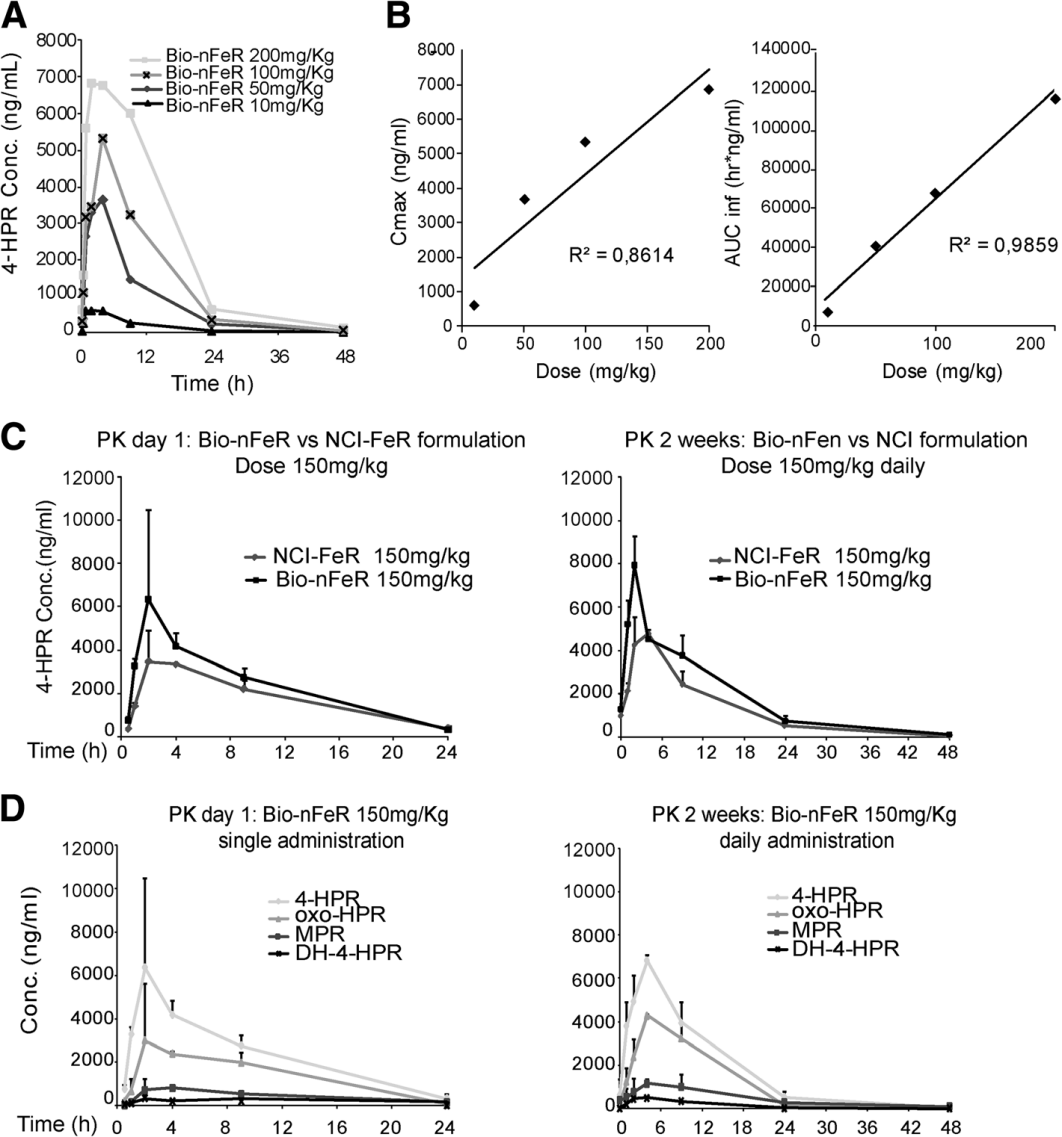
(A-B) *Plasma pharmacokinetic profiles of fenretinide after single oral administration of different Bio-nFeR doses*. **a** Comparison of the fenretinide plasma concentration decay curves after administration of the indicated Bio-nFeR oral doses. **b** Cmax and AUC of fenretinide as function of the administered dose of Bio-nFeR. **c**-**d** *Pharmacokinetic profile in acute and chronic administration of Bio-nFeR compared to NCI-FeR.* **c** Pharmacokinetic profile of oral Bio-nFeR in comparison with fenretinide NCI formulation after acute treatment (left) or 2 weeks of chronic treatment (right). **d** Pharmacokinetic profile of oral Bio-nFeR and its metabolites as single administration or chronic treatment and its metabolites in the same samples as in C

As shown in Fig. 2a, after oral administration of different doses of Bio-nFeR to mice, 4-HPR achieves plasma Cmax between 2 and 4 h, it is distributed rapidly and eliminated with half-life of about 7 h, warranting a drug plasma exposure up to 48 h at each dose investigated.

The main derived pharmacokinetic parameters are listed in Table 1: Cmax and AUC increased proportionally with the doses, indicating that the pharmacokinetic of Bio-nFeR is not dose dependent in the range of doses studied as shown in Fig. 2b that reports the correlations between dose vs Cmax and vs AUC. These results suggest that drug dose could be increased further to reach superior plasma exposure, eventually enhancing the therapeutic effect.

**Table 1:** Bio-nFeR pharmacokinetic parameters

Bio-nFeR pharmacokinetic parameters				
Bio-nFer Dose (mg/kg)	10	50	100	200
Cmax (ng/ml)	600.9	3654.7	5340.9	6837.8
Tmax (hr)	2	4	4	2
AUC 0-last (hr*ng/ml)	6745.4	39156.9	66323.6	112957.1
AUC inf (hr*ng/ml)	6812.1	39513.7	66751.7	114227.6
HL (hr)	7.48	7.32	6.49	7.16

Pharmacokinetic parameters analysis in mice after single oral treatment of 4 doses of Bio-nFeR: 10, 50, 100 and 200 mg/ /kg. Cmax: maximum plasma concentration achieved after drug administration; Tmax: time until Cmax is reached; AUC last: experimental area under the concentration-time curve from time 0 to the last experimental point measured (Clast); AUC inf: AUC calculated from 0 to Clast added of the extrapolated portion of the AUC calculated by: AUCz = Clast/ke; HL: plasma half-life of the terminal phase calculated by: HL = 0.693/ke.

Following the pharmacokinetic study of different doses as acute treatment, pharmacokinetic was investigated in an extensive comparative study, at therapeutic doses of Bio-nFeR and NCI-FeR. The two schedules of chronic administration were well tolerated in mice. No body weight loss or impaired liver function, were observed in treated- versus control-mice as carried out evaluating the hepatic enzymes levels (Additional file 1: Table S1). The schedules used are highly compatible with in vivo activity studies. 150 mg/Kg of Bio-nFeR and NCI-FeR were given as single administration (formally day 1) or for 2 weeks of daily administration (12 days on, 2 days off) and the plasma concentration-versus-time profiles of 4-HPR is shown in Fig. 2c. From a visual inspection of the curves and as indicated by the parameters listed in the Table 2, it can be seen that the exposure to 4-HPR is 1.3 and 1.6 times higher with oral Bio-nFeR than with oral NCI-FeR after the first treatment and after the repeated treatments, respectively.

**Table 2:** *Pharmacokinetic comparison of Bio-nFeR and NCI-Fer in single* versus *chronic treatment*

	4-HPR dose 150 mg/Kg			
	Single acute treatment		2 weeks of treatment daily	
Parameters	NCI-FeR	Bio-nFeR	NCI-FeR	Bio-nFeR
Cmax (ng/ml) SD	3456.8 ± 1453.3	6350 ± 4153.9	4306.4 ± 1150.7	6813.3 ± 1200.9
Tmax (hr)	2	2	2	4
AUC inf (hr*ng/ml)	46873.8	59371	53554.7	85378.7
AUC 24 h (hr.*ng/ml)	43257.7	56880.7	45436.7	78608.3
HL (hr)	6.4	5.3	8.3	5.7
R*	1.3		1.6	

Pharmacokinetic parameters determination in mice after single oral treatment of Bio-nFeR at dose of 150 mg/kg or after 2 weeks treatment, in comparison to the NCI-FeR formulation

In details, following the administrations of Bio-nFeR, 4-HPR reaches Cmax between 2 and 4 h, it is distributed rapidly and eliminated with a half life (HL) of about 5–6 h achieving adequate plasma exposure up to 48 h after the last administration. On day one, mean Cmax and AUC_0-24h_ were 6350 ng/mL and 56,881 ng/mL*h and after the repeated treatment 6813 ng/mL and 78,608 ng/mL*h. The last value indicates an increase of 40% of the plasmatic exposure of 4-HPR after chronic administration of Bio-nFeR in comparison with day one.

R* = AUC_inf_ ratio of repeated treatments vs acute treatment normalized per dose.

The elimination of fenretinide takes place mainly by metabolic process with formation of 4-oxo-4-HPR and MPR and via fecal excretion of the unmodified 4-HPR and metabolites.

As it can be seen in Additional file 1: Figure S1A reporting the plasma concentration-time profiles of the metabolites obtained in the study of multiple doses, 4-oxo-4-HPR and MPR in comparison to 4-HPR, amounted to about 50 and 15% after Bio-nFeR administration. Similar results were obtained for NCI-FeR administrations in comparative experiments of the two formulations (Additional file 1: Figure S1B). The main pharmacokinetic parameters obtained for Bio-nFer metabolites are reported in Additional file 1: Table S2A, comparison of single vs chronic Bio-nFer treatment in Fig. 2d and comparison of Bio-nFer vs NCI in Additional file 1: Table S2B.

A similar metabolic picture was seen during chronic administrations being the metabolites measurable in plasma 1 hour after administration and with maximal exposure at 2–4 h (Fig. 2d). The metabolites disappear with the same half-life of the parent compound (Additional file 1: Table S3). As seen in the acute study, the relative percent amount versus 4-HPR of 4-oxo-4-HPR, known to be 2–4-fold more cytotoxic than 4-HPR, and of the inactive metabolite MPR, after single and repeated treatment corresponded to about 50 and 15%, respectively. In addiction we detected the presence of a third metabolite, DH-4-HPR, previously described by Cooper et al. [4] that amounted to about 5–7% of the parental drug. As last consideration on metabolites formation, no significant differences were recorded in their plasma AUC between day 1 and day 14 (the metabolites AUCs increase, but reflect in a proportional way the increase of AUC of the parent drug), thus indicating that no phenomena of saturation or induction of the metabolism are present after the chronic treatment (Additional file 1: Table S3).

The fecal excretion of the parent drug and metabolites is reported in Additional file 1: Table S4. After both studies, the acute treatment at multiple doses and the chronic one, the proportion of Bio-nFeR and NCI-FeR eliminated in faeces as unmodified 4-HPR is comprised in the ranges 2–9%, for 4-oxo-4-HPR 4–33%, for DH-4-HPR 1–12% and less than 0.1% for MPR.

The amount of dose recovered in urine is negligible (≤0.002%) in line with the low polarity of fenretinide and metabolites (data not shown).

### Bio-nFeR exerts increased toxicity against lung CSC in vitro and exhibits improved plasma exposure

**Fig. 3:**
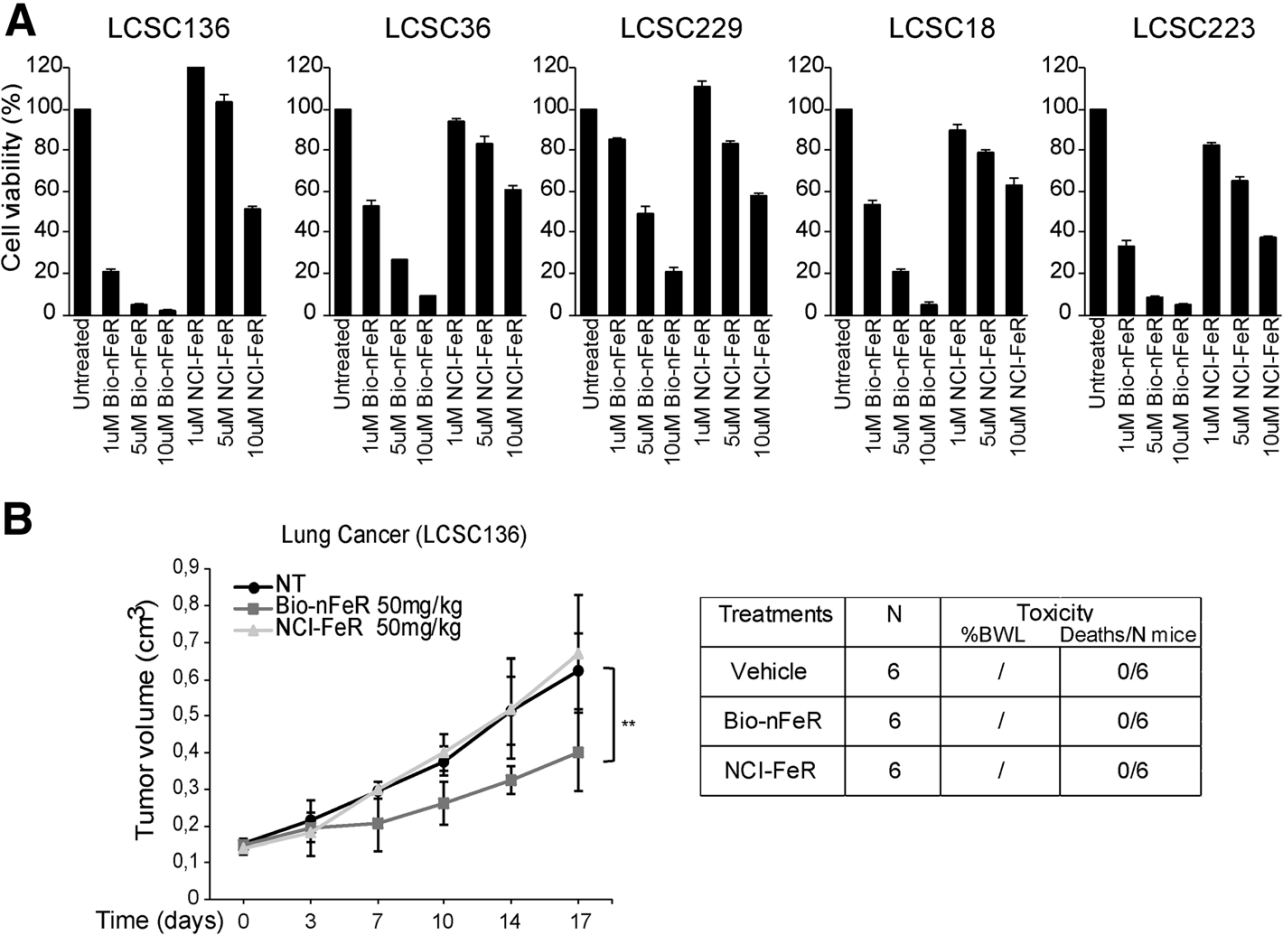
(**a**) *Cytotoxic activity of Bionanofenretinide (Bio-nFeR) in comparison with standard formulation fenretinide (NCI-FeR) in lung CSC*. Lung CSC were exposed to the indicated drug doses and cell viability was evaluated by CellTiter-Glo after 72 h and indicated as percentage versus control cells. **b** *Bio-nFeR and NCI-FeR antitumour activity in lung CSC-derived xenografts*. (left) Growth curves of lung CSC-derived xenografts in control mice or mice treated with Bio-nFeR or NCI-FeR at 50 mg/kg dose for the indicated times. Lung CSC sample #136 was used for xenograft generation. Mean ± S.D. of three independent experiments is shown. **P < 0.01. (right) Table of drug-induced systemic toxicity in the three groups of mice indicated as percentage of body weight loss (BWL) or number of deaths/total number of mice

With the aim to determine whether Bio-nFeR was endowed with valuable antitumour activity, its ability to affect lung-CSC viability was evaluated in comparison with the standard oral capsule formulation of fenretinide (NCI-FeR). Five previously isolated and validated patient-derived lung-CSC of different subtypes (Squamous Cell Carcinoma, Adenocarcinoma and Large cell Carcinoma) [44], were exposed to three different doses of Bio-nFeR or in parallel to NCI-FeR at the same concentrations. Bio-nFeR exerted a substantial cytotoxic effect, against all lung-CSC examined even at low concentrations although with variable extent in different samples, while more than 95% cells were killed at higher doses in all samples tested. In contrast, NCI-FeR determined a moderate (40–60%) reduction in cell viability only at higher doses (10 μM). More precisely, Bio-nFeR inhibited cell viability to a comparable extent than that determined by 10 fold higher concentration of NCI-FeR within the drug doses range tested, as visible in MTT assay-derived viability curve. Moreover, these finding was confirmed by the IC_50_ values calculated through a comparative dose response experiment performed on lung CSC that evidenced a 10 fold difference in IC50 with Bio-nFeR IC_50_ = 0.72 μM and NCI-FeR IC_50_ = 9 μM (Fig. 3a and Additional file 1: Figure S2).

We next examined the antitumour activity of Bio-nFeR in lung-CSC derived xenografts obtained in NSG immunodeficient mice, in comparison with the standard NCI-FeR formulation. While NCI-FeR administration (50 mg/kg) did not substantially affect tumour growth rates compared to tumours of untreated mice, the same dose of Bio-nFeR determined a significant inhibition of tumour growth (Fig. 3b left). Importantly, no signs of systemic toxicity (Fig. 3b right) were noticed suggesting that increased Bio-nFeR doses could be safely explored for future experiments, with the aim to further enhance efficacy.

### Bio-nFeR displays broad antitumour activity in vitro and in vivo, is well tolerated and reaches elevated and pharmacologically active intra-tumour concentrations

**Fig. 4:**
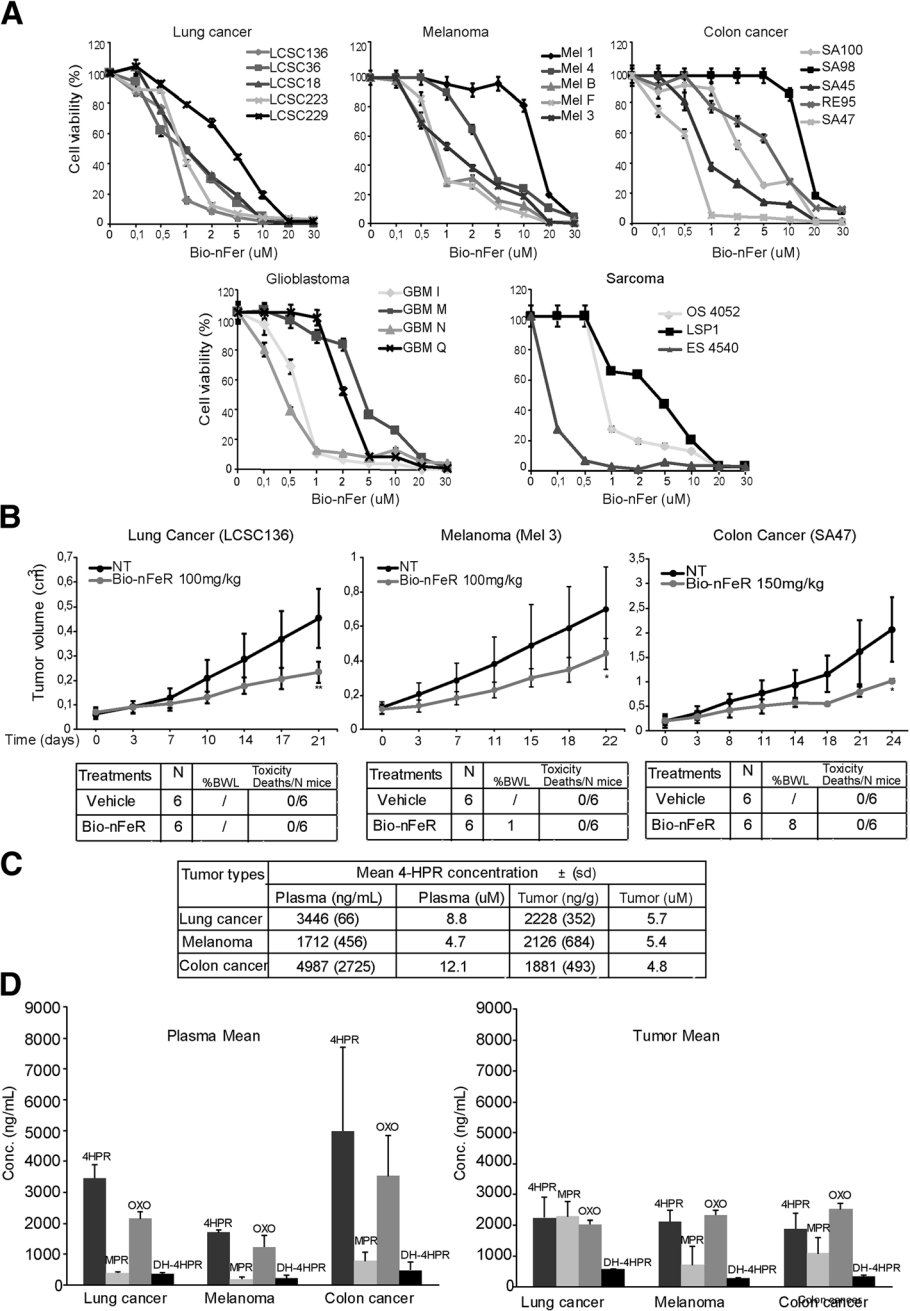
(**a**) *Cytotoxic activity of different doses of Bio-nFeR in lung cancer, melanoma, colon cancer, glioblastoma and sarcoma CSC* in vitro. The indicated CSC were exposed to 0.1, 0.5, 1, 2, 5, 10, 20 and 30 μM drug doses and cell viability was evaluated by Cell Titer-Glo after 72 h and indicated as percentage versus control cells. **b** *Bio-nFeR antitumour activity in lung cancer, melanoma and colon cancer CSC-derived xenografts*. (Upper panels) Growth curves of CSC-derived xenografts in control mice or mice treated with Bio-nFeR at 100 (lung cancer and melanoma) or 150 (colon cancer) mg/kg dose for the indicated times. Mean ± S.D. of three independent experiments is shown. *P < 0.05 **P < 0.01. (lower panels) Table of drug-induced systemic toxicity in the three groups of mice indicated as percentage of body weight loss (BWL) or number of deaths/total number of mice. **c** *Fenretinide levels in plasma and tumours of the same samples as in B.* Fenretinide concentration in plasma and tumours is expressed in ng/ml and the corresponding μM concentration, as indicated (tumour density is assumed as approximately =1). **d** *Fenretinide metabolites levels in plasma and tumours of the same samples as in B-C.* Fenretinide, 4HPR, OXO-4HPR and DH-4HPR concentration levels in the indicated plasma and tumours of the same samples as in C

Given the marked antitumour activity of Bio-nFeR against lung cancer cells in vitro and in vivo and the enhanced systemic exposure compared to the reference fenretinide formulation, we next extended the evaluation of its cytotoxic activity in a wide range of concentrations against lung, colon cancer, melanoma, sarcoma and glioblastoma CSC. Three days exposure to Bio-nFeR determined a marked inhibition of CSC viability in the majority of CSC samples of each tumour type tested (Fig. 4a). Generally, while glioblastoma and sarcoma-CSC lines resulted more resistant to Bio-nFer treatment, a high fraction of lung, colon and melanoma CSC lines displayed promising responsiveness to Bio-nFeR, prompting subsequent in vivo studies to assess the drug antitumour activity in the corresponding xenograft models. Subcutaneous CSC-derived lung, melanoma and colon tumours were generated in immunodeficient mice and Bio-nFeR was orally administered for 3 weeks at 100 (lung cancer and melanoma) or 150 (colon cancer) mg/kg, based on the absence of toxicity demonstrated in the previous experiment (Fig. 3b), where lower doses of drug were used. Tumours of treated mice showed a prominent reduction of the tumour growth rate, compared to control tumours (Fig. 4b)*.* Moreover, the drug displayed high tolerability, as no systemic toxicity was observed, except for a minor (8%) weight loss in the higher-dose mice group (150 mg/kg), in absence of other visible signs of toxicity (Fig. 4b bottom panels). The lack of mice toxicity, together with acceptable patient tolerability observed when 10 times higher doses have been administered in clinical trials, suggests that the maximum tolerated dose has not been achieved under these experimental conditions and can be safely escalated to further enhance drug amount at the tumour site and therapeutic efficacy [13].

In line with its antitumour efficacy, described above, the parallel pharmacokinetic study showed that fenretinide reached highly encouraging concentrations in plasma (Fig. 4c), higher than that reached in plasma of patients treated with equivalent doses (300 mg/m^2^ is the equivalent dose corresponding to 100 mg/kg in mice) of the standard drug or of other improved drug formulations in clinical trials, both as single or multiple administrations [20, 27].

Importantly, as measured here for the first time in tumour models, the drug showed pharmacologically active intra-tumour concentration (ranging from 1.88 μg/g to 2.23 μg/g, equivalent to 4.8–5.7 μM) at both drug doses analyzed (Fig. 4c). These concentrations were superior to those required for cytotoxicity in vitro in these cell lines (Fig. 4a).

In the tumours analyzed, the three main metabolites of 4-HPR already seen in plasma were detected (Fig. 4d). The most abundant was 4-oxo-4-HPR, the active one, that achieved comparable tumour concentrations in the three different models. These concentrations were in the same range of those of the parent drug, but since this metabolite is 2–4 fold more active than 4-HPR, we can speculate that the in vivo antitumour activity is mainly due to its conspicuous presence (Additional file 1: Table S5).

### Bio-nFeR antitumour activity is associated with reduced tumour cell proliferation, apoptosis induction, modulation of lipid metabolism and decrease of CSC features

**Fig. 5:**
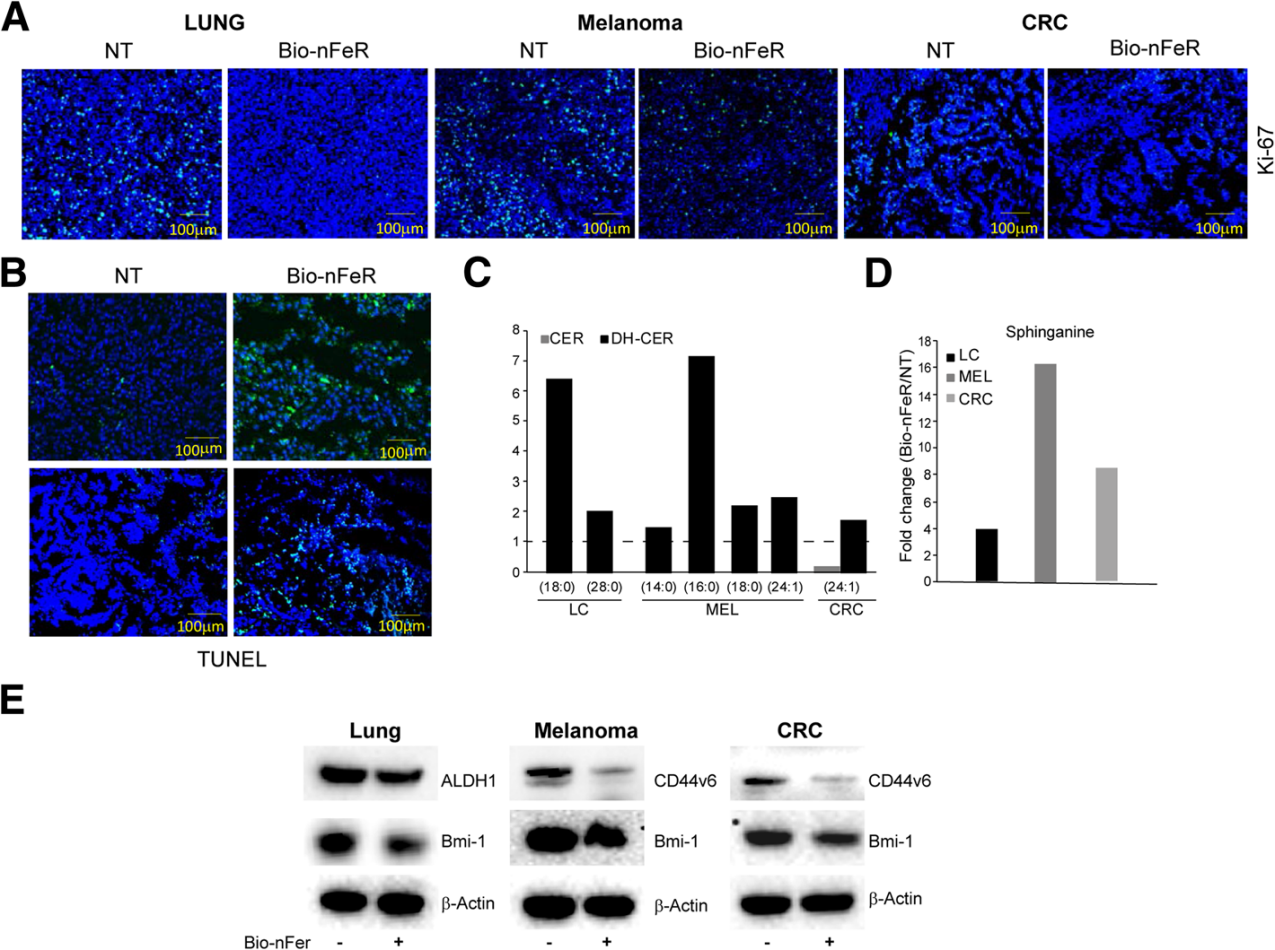
*Reduction of tumour cell proliferation, apoptosis induction and modulation of lipid metabolism by Bio-nFeR.* **a** Confocal images of KI-67 immunofluorescence of tissue slides from control or Bio-nFeR treated lung cancer, melanoma and colorectal (CRC) cancer xenografts. **b** TUNEL assay, showing the levels of apoptosis induction at both doses used (upper panels correspond to 100 mg/kg-treated lung cancer xenografts and lower panels to 150 mg/kg-treated colon cancer xenografts). **c** Individual dihydroceramides composition of lung (LC), melanoma (MEL) and colorectal (CRC) tumours treated with Bio-nFeR at single 100–150 mg/Kg administration were identified by acyl chain, normalized to control and plotted as fold change. **d** The amount of sphinganine in the same samples as in A and B, normalized to control and plotted as fold change. **e** Immunoblot analysis of CSC antigens ALDH1, CD44V6 and Bmi-1 in the indicated control (−) or Bio-nFeR treated (+) xenografts. β-actin blot was used for equal loading control

To dissect the molecular effects of Bio-nFeR treatment in vivo, control and treated tumour xenografts were analyzed for their proliferative index and extent of cell death induction. As clearly visible in confocal images of tumour tissues slides in Fig. 5a, the expression of KI-67 was strongly reduced in all treated tumours. Moreover, tumours treated with Bio-nFeR at both doses of 100 and 150 mg/kg displayed a markedly increased fraction of TUNEL-positive cells in comparison with controls (Fig. 5b). Thus, Bio-nFeR induced a marked inhibition of tumour cell proliferation associated with apoptosis induction, in line with previous reports [2, 8].

Previous reports showed that fenretinide-mediated cytotoxicity is associated with dysregulation of lipid metabolism. Specifically, fenretinide is able to increase the intracellular levels of dihydroceramides species by targeting the dihydroceramide desaturase [16, 29, 38, 51]. Liquid chromatography-mass spectrometry (LC-MS), showed that Bio-nFeR treatment induced a marked increase of dihydroceramide and dihydrosphingolipids (glucosyldihydroceramide) species (Fig. 5c and Additional file 1: Figure S3). This effect is more evident for dihydroceramides species with 18 and 16 fatty-acid chains. Conversely, saturated ceramides levels were not affected by Bio-nFeR treatment. In concomitance with the accumulation of long-chain dihydroceramide, we also observed a significant increase of the intracellular levels of sphinganine in Bio-nFeR-treated tumour xenografts (Fig. 5d). Based on our previous results, we next confirmed that xenografts derived from the specific CSC lines used were heterogeneous and composed by a minor sub-population of CSC within a majority of differentiated tumour cells [42, 47, 50]. In fact, flow cytometry analysis for CSC specific markers confirmed that while aldefluor+ (lung) or CD44V6+ (melanoma and colon) cells constituted a large fraction of the CSC culture population in vitro as expected for a CSC-enriched population, this fraction dramatically decreased in the corresponding xenografts (Additional file 1: Figure S4A). Thus, to determine whether Bio-nFeR treatment was effective against CSC within the tumour, we analyzed the CSC features of control or Bio-nFeR treated tumour xenografts, based on the expression of CSC-restricted antigens suitable for each tumour type [7, 9, 21, 41, 42, 44, 46, 47, 50]. We found that xenografts derived from mice treated with Bio-nFeR displayed decreased expression of CSC-specific antigens compared to control tumour, demonstrating that drug treatment determined a preferential CSC targeting within the tumour. Thus, Bio-nFeR tumours displayed decreased CSC features, implying decreased aggressiveness as compared with control tumour (Fig. 5e and Additional file 1: Figure S4B).

**Fig. 6:**
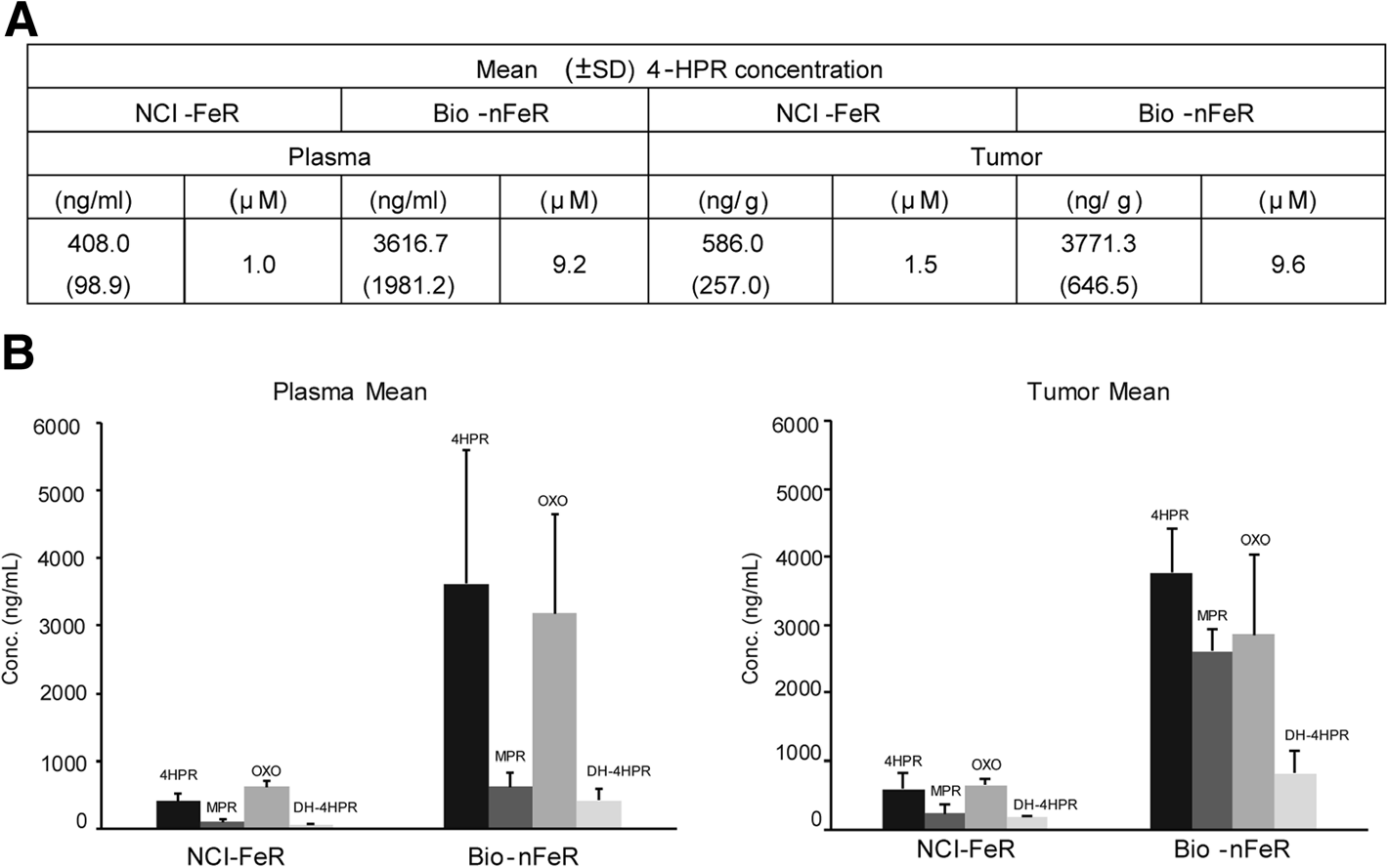
Comparison *of fenretinide levels in blood and tumours of mice treated with standard NCI-FeR or Bio-nFeR formulations.* **a** Fenretinide concentration (± standard deviation) in plasma (expressed in ng/ml and the corresponding μM concentration are indicated) and tumours (in ng/g and the extrapolated μM concentration assuming tumour density as approximately = 1) of mice after 3 weeks 100 mg/kg oral administration of each drug formulation. **b** Fenretinide metabolites levels in plasma and tumours of the same samples as in A. Fenretinide, 4HPR, OXO-4HPR and DH-4HPR concentration levels in the indicated plasma and tumours of the same samples as in A

As we observed that Bio-nFeR was superior than NCI-FeR at 50 mg/kg dose (Fig. 3) in terms of anti tumour activity and that increased HPR concentration was detected in plasma of Bio-nFeR treated mice compared to NCI-FeR treated animals at higher therapeutic doses (Fig. 2), we next evaluated whether at the dose of 100 mg/kg, therapeutically active for Bio-nFeR, NCI-FeR formulation could reach therapeutic drug levels within tumour. In a comparative study, in which tumour-bearing mice were treated with the two alternative drug formulations at 100 mg/kg dose, Bio-nFeR confirmed superior antitumour activity compared to NCI-FeR (Additional file 1: Figure S5), as well as higher plasma bioavailability (Fig. 6a). Moreover, in line with these results, Bio-nFeR treatment determined much higher intra tumour concentration of 4-HPR and its active metabolite 4-oxo-4-HPR, compared to that reached following NCI-FeR administration (Fig. 6), demonstrating the higher tropism of Bio-nFeR also at the tumour level, in line with its superior anti tumour activity.

## Discussion

Cellular and molecular heterogeneity of tumours represents an obstacle for the achievement of long-term therapeutic efficacy. Cell fractions endowed with therapy-resistant stem cell-associated features are represented by CSC, which are themselves a heterogeneous population characterized by epigenetic and functional traits rather than specific molecular alterations [6]. Thus, tumour diversity needs to be accurately considered, to become an option for cancer therapy. For instance, specific CSC-properties can be targeted to achieve durable effects and the efficacy of molecularly-targeted compounds needs to be validated in the CSC compartment to assess its long-term therapeutic benefit. Furthermore, another issue is the necessity to reach therapeutic drug concentrations at the tumour site to increase drug activity against the more resistant CSC while reducing systemic toxicity. This aim can be accomplished through targeted-delivery of drugs to the tumour site or through appropriate drug modifications favoring accumulation at the target site, limiting drug degradation in the blood or increasing its solubility in body fluids and in tumour microenvironment.

Here, we explored the activity of a promising anti-tumour agent, fenretinide, against CSC of different tumours upon enhancing its antitumour activity through a nanocarrier-based approach. Fenretinide has been previously proved to be a promising anticancer agent with ability to affect CSC-related properties and viability of cell lines of different tumours including medulloblastoma, melanoma, breast and myeloma cell lines [1, 10, 33, 53]. In contrast, fenretinide activity in vivo is severely hampered by its hydrophobic character which restrains its bioavailability and prevents the achievement of therapeutic drug levels in the body compartments. Thus, due to fenretinide potential cytotoxic activity against aggressive tumour cells and in view of its proved negligible systemic toxicity, drug development has been fostered through drug formulations aimed at improving its solubility and bioavailability. Encouraging results have been obtained in this direction, including a fenretinide-cyclodextrin complex developed in our laboratory which, however, required intravenous administration [36] ([4, 8, 20]; B. J. [27, 32, 37]).

We developed a new nano-micellar fenretinide formulation called bionanofenretinide (Bio-nFeR), based on ion pair formation between fenretinide and phosphatidylcholine. Such pairing triggers the spontaneous formation in water of micelles, containing a fenretinide inner core and an hydrophilic shell formed by the spontaneous assembling of the phospholipid molecules. The nanomicelles were designed to raise fenretinide solubility and bioavailability by oral administration and provide drug levels in the body compartments suitable to elicit a therapeutic response. To assess the therapeutic potential of the Bio-nFeR formulation we compared its activity in vitro and in tumour xenografts with that of the standard formulation made of soft gelatin capsules containing fenretinide in corn oil and polysorbate, which is currently available at the National Cancer Institute (NCI-FeR) and has been largely tested in clinical trials [13]. Bio-nFeR displayed encouraging cytotoxic activity against all lung-CSC tested and its activity was superior than the standard NCI-FeR, with more than 10 fold difference in the IC_50_ in most cases (Fig. 2a and Additional file 1: Figure S2). Enhanced antitumour activity was observed in vivo*,* as Bio-nFeR proved to be far more effective in the growth inhibition of xenografts generated by lung-CSC (Fig. 4). Importantly, no signs of systemic toxicity were noticed, in line with the high tolerability described for patients treated with comparable doses of different formulations in clinical trials and suggesting that increased Bio-nFeR doses could be safely explored for future experiments, with the aim to further enhance its efficacy. In line with robust activity in vivo*,* Bio-nFeR displayed an improved pharmacokinetic profile than NCI-FeR. In fact, in contrast to NCI-FeR that showed a dose dependent pharmacokinetic curve, the pharmacokinetics of Bio-nFeR was not dose dependent and appeared linear in the doses range studied (Fig. 2c). These observations suggest that drug dose could be further increased to reach superior plasma exposure and enhance the therapeutic effect.

Besides lung cancer, Bio-nFeR cytotoxic activity was evaluated in a wide range of patient-derived CSC samples of different origin including melanoma, glioblastoma, colon cancer and sarcoma. In the context of a general marked efficacy, only a minority of CSC samples resulted less sensitive to Bio-nFeR suggesting that the drug is endowed with broad and prominent CSC-efficacy, even though biomarkers of response might be helpful to identify the responsive cells. The more promising drug activity was observed for lung, colon and melanoma tumour-derived CSC and the corresponding CSC-derived xenografts displayed high sensitivity to Bio-nFeR treatment (Fig. 4). Based on the lack of toxicity observed in the first experiments drug doses were escalated up to 100–150 mg/kg, determining improved efficacy with negligible side effects. Bio-nFeR antitumour activity was associated with reduced tumour cell proliferation, apoptosis induction and modulation of lipid metabolism at both Bio-nFer doses used (Fig. 5). The fenretinide doses used in mice experiments are comparable to the lower range doses used in drug escalation clinical trials using NCI-FeR or other fenretinide formulations, accordingly to the body surface area conversion method used to calculate the human equivalent doses (HED), as previously reported [39]. In fact, as 100 mg/kg (dose used in mice) is equivalent to 300 mg/m2, and up to 10 times higher doses were tolerated in clinical trial patients, it is likely that the drug dose of Bio-nFeR can be escalated further in absence of substantial side effects and the therapeutic efficacy correspondingly increased (the maximum dose used is pediatric cinical trial has been 4000 mg/m2 and displayed manageable toxicity) [13, 15].

Our pharmacokinetics studies showed that single Bio-nFeR administration at 100 mg/kg/daily determined high plasma concentrations, higher than the therapeutic concentrations and superior to those found in other mice studies after both single or chronic administration of the standard fenretinide formulation (NCI-FeR) or with the more recently introduced oral powder lipid matrix formulation, called LYM-X-SORB matrix (13.64 μM plasma concentration was obtained after a single 100 mg/kg Bio-nFeR administration, versus 1.6 μM reported for NCI-FeR and 5.1 μM for LYM-X-SORB after repeated administrations of 120 mg/kg for 4.5 days) (Barry J. [26]).

Thus, the oral bioavailability has been found to be acceptable and reproducible in the doses range studied. Bio-nFeR has showed, at therapeutic doses of fenretinide, higher systemic exposure than that obtained with the reference NCI formulation, especially during the chronic pharmacokinetic study conducted after 1 or 2 weeks of daily administration. The high bioavailability contributed to reach unexpectedly high drug concentrations within tumour tissue in three different models tested (lung, colon and melanoma) after mice treatment with Bio-nFeR (Fig. 4c, d). In fact, the new formulation showed intratumour concentration of fenretinide pharmacologically active, superior to those required for cytotoxicity in vitro (Figs. 2a and 3). Finally, intratumour concentration of 4HPR obtained following Bio-nFeR treatment was much higher than that obtained with the standard NCI-FeR administered at the same dose. These results enforced the assumption that the increased antitumour efficacy of Bio-nFeR in vivo, compared to NCI-FeR, is the direct consequence of the improved bioavailability obtained with the new formulation that achieved higher drug concentration at tumour site.

## Conclusions

In conclusion, with the aim to explore the possibility of fully exploit the therapeutic potential of fenretinide, we generated and tested, for the first time in in vitro and in vivo models, the anti-tumour activity of fenretinide nanoencapsulated in a new micellar system, Bio-nFeR, designed to improve its bioavailability and obtain effective anti-tumour plasma concentrations. Bio-nFeR therapeutic efficacy was superior to previous formulations and displayed negligible systemic toxicity at therapeutic doses. Our results propose Bio-nFeR as a CSC-effective innovative formulation of fenretinide with increased antitumour activity in vitro and in vivo due to improved drug solubility and bioavailability, that guarantees therapeutic drug exposure at tumour site in the absence of toxicity. Our results strongly encourage further preclinical-investigation on Bio-nFer in solid tumour CSC in the direction of clinical development.

## Additional file

Additional file 1: **Figure S1.** Metabolites plasma concentrations (MPR and oxo-4-HPR) determined in mice after single oral treatment of 4 doses of Bio-nFeR: 10, 50, 100 and 200 mg/Kg. The analysis was performed after 0, 24, 48 h. **Figure S2.** Evaluation of the 50% inhibitory concentration (IC50) of BionFeR and NCI-FeR formulation was done on a representative lung cancer stem cell line (LCSC). The cells were exposed to the indicated drug doses (0–30 μM) and cell viability was evaluated by CellTiter-Glo after 72 h. **Figure S3.** A) Sphingomyelin-Ceramide (SM-CER) and Sphingomyelin- Dihydro-Ceramide (SM-DH-CER) content in lung (LC), melanoma (MEL) and colon (CRC) tumors treated with Bio-nFeR at single 100–150 mg/Kg administration were quantified through Ultra High Performance Liquid Chromatography (UHPLC), normalized to control and plotted as fold change. B) Quantification of Glucosyl-Ceramide (GLC-CER) and Glucosyl-Dihydro-Ceramide (GLC-DH-CER) in the same samples as in A, normalized to control and plotted as fold change. **Figure S4.** Cancer stem cell marker expression in CSC-culture conditions and in CSC-derived xenografts. A) Flow cytometry analysis of Aldefluor in (left panels) and CD44v6 (middle and right panels) in the indicated CSC and xenograft samples. B) Immunoblot analysis of CSC antigens c-Kit and SOX-2 in the indicated control (−) or BionFeR treated (+) melanoma xenografts. β-actin blot was used for equal loading control. **Figure S5.** Growth curves of lung CSC-derived xenografts in control mice or mice treated with Bio-nFeR or NCI-FeR at 100 mg/kg dose for the indicated times. Mean ± standard error is shown. *P < 0.05. **Table S1.** Hepatic enzyme determination in mice after single administration or 2 weeks oral daily treatment at dose of 150 mg/kg of Bio-nFeR or NCIFeR formulation. AST: Aspartate transaminase; ALT: Alanine transaminase; Bil tot: Bilirubin total; Vehicle after 24 h. **Table S2.** Quantification of 4-HPR and metabolites (oxo-4-HPR e MPR) determined in mice after oral Bio-nFeR single treatment of BNF: 10, 50, 100 and 200 mg/Kg (A) or Bio-nFer at dose of 200 mg/kg in comparison to the NCI-FeR formulation (B). Cmax: maximum plasma concentration achieved after drug administration; Tmax: time until Cmax is reached; AUC last: experimental area under the concentration-time curve from time 0 to the last experimental point measured (Clast); HL: plasma half-life of the terminal phase calculated by: HL = 0.693/ke. **Table S3.** 4-HPR and metabolites (oxo-4-HPR, MPR and DH-4-HPR) evaluated after oral Bio-nFeR single (day 1) or repeated daily treatment at dose of 150 mg/kg. Cmax: maximum plasma concentration achieved after drug administration; Tmax: time until Cmax is reached; AUC last: experimental area under the concentration-time curve from time 0 to the last experimental point measured (Clast); HL: plasma half-life of the terminal phase calculated by: HL = 0.693/ke. T**able S4.** 4-HPR and metabolites concentrations measured after 24 h in feces of mice after acute treatment at different doses of Bio-nFeR (10, 50, 100 and 200 mg/Kg) (A) and after chronical treatment at dose of 150 mg/kg (B), in comparison with the NCI-FeR formulation. **Table S5.** 4-HPR and metabolites (MPR, oxo-4-HPR and DH-4-HPR) concentrations in plasma and tumors (lung cancer, melanoma and colon cancer) as in Fig. 4 BC. (PDF 308 kb)

## Data Availability

The datasets generated and/or analysed during the current study are available from the corresponding author on reasonable request.

## Abbreviations

4-HPR: 4-hydroxy(phenyl)retinamide)
Bio-nFeR: Bionanofenretinide
BWL: body weight loss
CRC: colorectal cancer
CSC: cancer stem cells
FeR: fenretinide
HED: human equivalent doses
LC: lung cancer
MEL: melanoma
NCI-Fer: National Cancer Institute fenretinide
NSG: NOD/SCID non-obese diabetic/severe combined immunodeficiency gamma chain deficient
TUNEL: Terminal deoxynucleotidyl transferase dUTP nick end labeling
