# Supplementary material for: A novel oral micellar fenretinide formulation with enhanced bioavailability and antitumour activity against multiple tumours from cancer stem cells
Source: J Exp Clin Cancer Res. 2019 Aug 22;38:373. doi: 10.1186/s13046-019-1383-9 (PMC6706930; doi:10.1186/s13046-019-1383-9)
Supplement: Supplementary file 1 — Figure S1. Metabolites plasma concentrations (MPR and oxo-4-HPR) determined in mice after single oral treatment of 4 doses of Bio-nFeR: 10, 50, 100 and 200 mg/Kg. The analysis was performed after 0, 24, 48 h. Figure S2. Evaluation of the 50% inhibitory concentration (IC50) of BionFeR and NCI-FeR formulation was done on a representative lung cancer stem cell line (LCSC). The cells were exposed to the indicated drug doses (0–30 μM) and cell viability was evaluated by CellTiter-Glo after 72 h. Figure S3. A) Sphingomyelin-Ceramide (SM-CER) and Sphingomyelin- Dihydro-Ceramide (SM-DH-CER) content in lung (LC), melanoma (MEL) and colon (CRC) tumors treated with Bio-nFeR at single 100–150 mg/Kg administration were quantified through Ultra High Performance Liquid Chromatography (UHPLC), normalized to control and plotted as fold change. B) Quantification of Glucosyl-Ceramide (GLC-CER) and Glucosyl-Dihydro-Ceramide (GLC-DH-CER) in the same samples as in A, normalized to control and plotted as fold change. Figure S4. Cancer stem cell marker expression in CSC-culture conditions and in CSC-derived xenografts. A) Flow cytometry analysis of Aldefluor in (left panels) and CD44v6 (middle and right panels) in the indicated CSC and xenograft samples. B) Immunoblot analysis of CSC antigens c-Kit and SOX-2 in the indicated control (−) or BionFeR treated (+) melanoma xenografts. β-actin blot was used for equal loading control. Figure S5. Growth curves of lung CSC-derived xenografts in control mice or mice treated with Bio-nFeR or NCI-FeR at 100 mg/kg dose for the indicated times. Mean ± standard error is shown. *P < 0.05. Table S1. Hepatic enzyme determination in mice after single administration or 2 weeks oral daily treatment at dose of 150 mg/kg of Bio-nFeR or NCIFeR formulation. AST: Aspartate transaminase; ALT: Alanine transaminase; Bil tot: Bilirubin total; Vehicle after 24 h. Table S2. Quantification of 4-HPR and metabolites (oxo-4-HPR e MPR) determined in mice after oral Bio-nFeR s [file 13046_2019_1383_MOESM1_ESM.pdf]

SUPPLEMENTARY FIGURE LEGENDS:

Supplementary Figure 1: Metabolites plasma concentrations (MPR and oxo-4-HPR) determined in mice after single oral treatment of 4 doses of Bio-nFeR: 10, 50, 100 and 200 mg/Kg. The analysis was performed after 0, 24, 48 h.

Supplementary Figure 2: Evaluation of the 50% inhibitory concentration (IC50) of Bio-nFeR and NCI-FeR formulation was done on a representative lung cancer stem cell line (LCSC). The cells were exposed to the indicated drug doses (0-30 μM) and cell viability was evaluated by CellTiter-Glo after 72 hours.

Supplementary Figure 3: A) Sphingomyelin-Ceramide (SM-CER) and Sphingomyelin-Dihydro-Ceramide (SM-DH-CER) content in lung (LC), melanoma (MEL) and colon (CRC) tumors treated with Bio-nFeR at single 100-150 mg/Kg administration were quantified through Ultra High Performance Liquid Chromatography (UHPLC), normalized to control and plotted as fold change. B) Quantification of Glucosyl-Ceramide (GLC-CER) and Glucosyl-Dihydro-Ceramide (GLC-DH-CER) in the same samples as in A, normalized to control and plotted as fold change.

Supplementary Figure 4: Cancer stem cell marker expression in CSC-culture conditions and in CSC-derived xenografts. A) Flow cytometry analysis of Aldefluor in (left panels) and CD44v6 (middle and right panels) in the indicated CSC and xenograft samples. B) Immunoblot analysis of CSC antigens c-Kit and SOX-2 in the indicated control (-) or Bio-nFeR treated (+) melanoma xenografts. β-actin blot was used for equal loading control.

Supplementary Figure 5: Growth curves of lung CSC-derived xenografts in control mice or mice treated with Bio-nFeR or NCI-FeR at 100 mg/kg dose for the indicated times. Mean± standard error is shown. *P<0.05

SUPPLEMENTARY TABLE LEGENDS:

Supplementary Table 1: Hepatic enzyme determination in mice after single administration or two weeks oral daily treatment at dose of 150 mg/kg of Bio-nFeR or NCI-FeR formulation. AST: Aspartate transaminase; ALT: Alanine transaminase; Bil tot: Bilirubin total; Vehicle after 24h.

Supplementary Table 2: Quantification of 4-HPR and metabolites (oxo-4-HPR e MPR) determined in mice after oral Bio-nFeR single treatment of BNF: 10, 50, 100 and 200 mg/Kg (A) or Bio-nFer at dose of 200mg/kg in comparison to the NCI-FeR formulation (B). Cmax: maximum plasma concentration achieved after drug administration; Tmax: time until Cmax is reached; AUC last: experimental area under the concentration-time curve from time 0 to the last experimental point measured (Clast); HL: plasma half-life of the terminal phase calculated by: HL = 0.693/ke,

***Supplementary Table 3*:** 4-HPR and metabolites (oxo-4-HPR, MPR and DH-4-HPR) evaluated after oral Bio-nFeR single (day 1) or repeated daily treatment at dose of 150 mg/kg. Cmax: maximum plasma concentration achieved after drug administration; Tmax: time until Cmax is reached; AUC last: experimental area under the concentration-time

curve from time 0 to the last experimental point measured (Clast); HL: plasma half-life of the terminal phase calculated by: HL = 0.693/ke,

Supplementary Table 4: 4-HPR and metabolites concentrations measured after 24 hours in feces of mice after acute treatment at different doses of Bio-nFeR (10, 50, 100 and 200 mg/Kg) (A) and after chronical treatment at dose of 150mg/kg (B), in comparison with the NCI-FeR formulation.

Supplementary Table 5: 4-HPR and metabolites (MPR, oxo-4-HPR and DH-4-HPR) concentrations in plasma and tumors (lung cancer, melanoma and colon cancer) as in Figure 4BC.

A

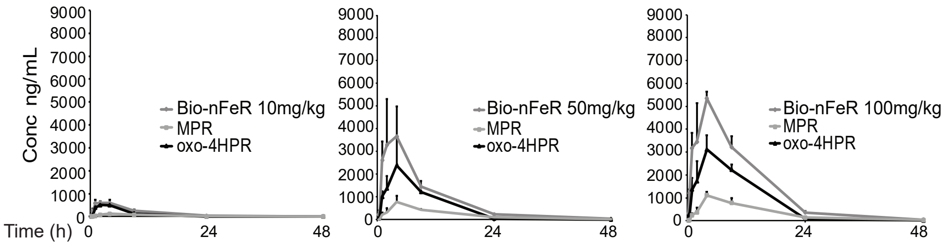

B

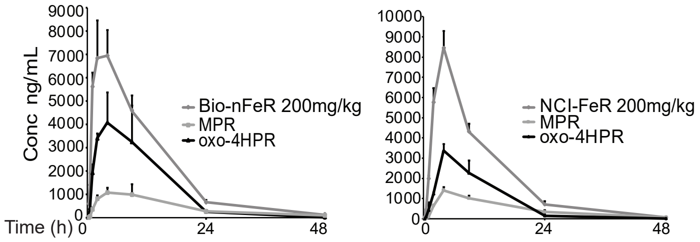

Supplementary Figure 1

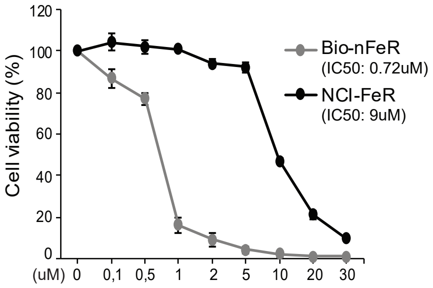

Supplementary Figure 2

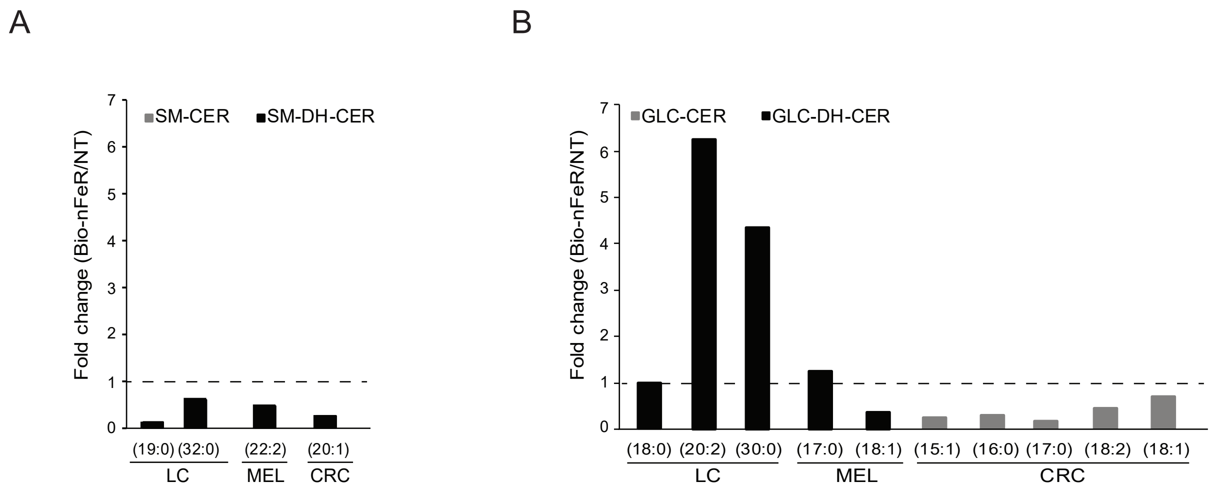

Supplementary Figure 3

A

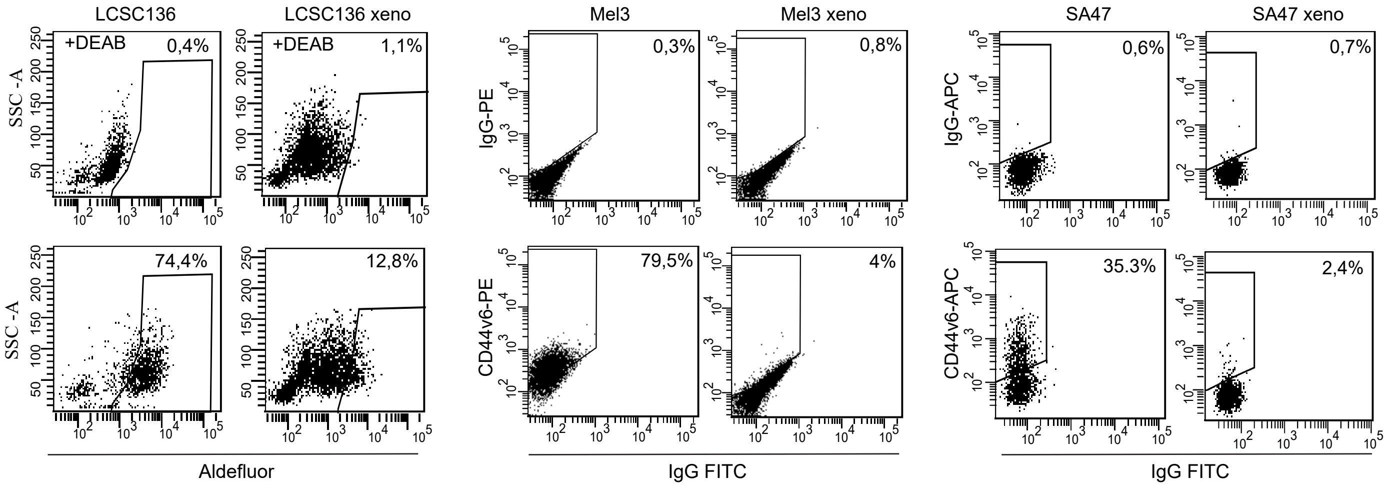

B

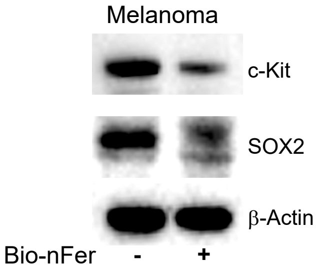

Supplementary Figure 4

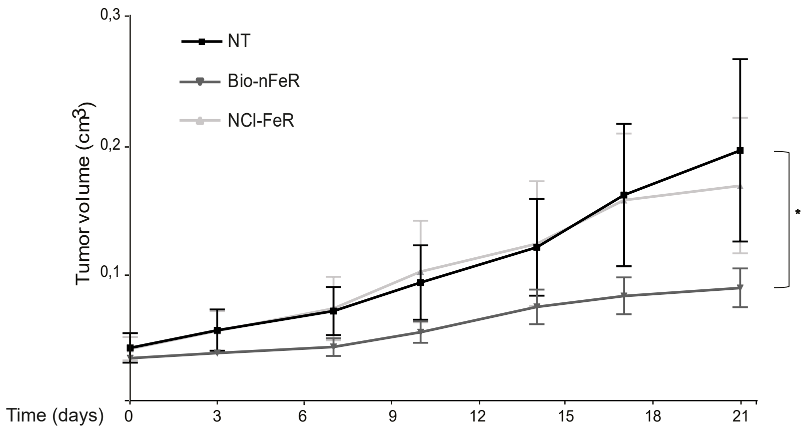

Supplementary Figure 5

Supplementary Table 1

Administration Schedule	Dose (mg/kg)	Formulation	AST		ALT		BIL_{tot}	
			24h	48h	24h	48h	24h	48h
Single admin.	150	NCI-FeR	123±35,64	-	38±1,73	-	0,39±0,11	-
		Bio-nFeR	120±39,34	-	34±4,58	-	0,49±0,01	-
2 wks 1 daily dose	150	NCI-FeR	85,6±8,2	137,66±32,25	28,3±3,05	32,66±5,5	0,4±0,07	0,65±0,36
		Bio-nFeR	96,3±2,51	108±28,58	30,3±3,78	30±6,24	0,31±0,12	0,82±0,27
Vehicle			89,3±8,14		26,7±4,93		0,4±0,05	

Supplementary Table 2 A

Bio-nFeR (mg/kg)	10	50	100	200	10	50	100	200	10	50	100	200	10	50	100	200
Parameters	Cmax ±SD (ng/ml)				Tmax (hr)				AUC 0-last (hr*ng/ml)				HL (hr)			
4-HPR	600.9 ± 68.1	3654.7 ±1312.9	5340.9 ±294.4	6837.8 ±1613.8	2	4	4	2	6745.4	39156.9	66323.6	112957.1	7.5	7.3	6.5	7.2
oxo-4-HPR	505.1 ±82.9	2389.1 ±1275.4	3109.1 ±618.1	4071.2 ±1295.5	2	4	4	4	4145.3	24372.6	38500.3	58677.2	5.2	5.4	4.8	5.3
MPR	120.6 ±23.8	778.9 ±273.2	1112.6 ±145.7	1070.5 ±216.7	4	4	4	4	1750.7	9931.5	15439.7	21449.6	9.4	10.3	8.6	11.6

Supplementary Table 2 B

	4-HPR dose 200mg/Kg							
	NCI-nFeR	Bio-nFeR	NCI-nFeR	Bio-nFeR	NCI-nFeR	Bio-nFeR	NCI-nFeR	Bio-nFeR
Parameters	Cmax ±SD (ng/ml)		Tmax (hr)		AUC 0-last (hr*ng/ml)		HL (hr)	
4-HPR	8463.7 ±826.0	6837.8 ±1613.8	4	2	97906.1	112957.1	6.7	7.2
oxo-4-HPR	3372.3 ±327.9	4071.2 ±1295.5	4	4	39875.4	58677.2	5.4	5.3
MPR	1407.0 ±162.6	1070.5 ±216.7	4	4	23406.1	21449.6	9.9	11.6

Supplementary Table 3

Parameters	Cmax ±SD (ng/ml)		Tmax (hr)		AUC 0-last (hr*ng/ml)		HL (hr)	
Dose (mg/kg)	Day 1<br>150	2 week<br>150 daily	Day 1<br>150	2 week<br>150 daily	Day 1<br>150	2 week<br>150 daily	Day 1<br>150	2 week<br>150 daily
4-HPR	6350.0<br>±4153.9	6813.3<br>± 1200.9	20	4	56880.7	85097.1	5.3	5.7
oxo-4-HPR	2979.4<br>± 2637.9	4282.6<br>±914.3	2.	4	33726.1	54434.2	3.9	4.7
MPR	798.0<br>±127.4	1147.9<br>165.7	4	4	10435.4	22086.1	8.6	11.2
DH-4-HPR	308.5<br>±245.7	483.8<br>± 97.1	2	4	3106.4	5964.8	3.9	5

Supplementary Table 4A

Fecal excretion of acute treatment at multiple doses

	4-HPR		MPR		oxo-4HPR		DH-4HPR	
Dose (mg/kg)	NCI-FeR	Bio-nFeR	NCI-FeR	Bio-nFeR	NCI-FeR	Bio-nFeR	NCI-FeR	Bio-nFeR
10	x	4.7 (3.2%)	x	0.03 (0.02 %)	x	47 (32.6 %)	x	18.3 (12.3%)
50	x	38.2 (4.0%)	x	0.5 (0.05 %)	x	253.8 (26.5%)	x	77.1 (8.2%)
100	x	33.6 (1.9%)	x	0.7 (0.04 %)	x	85.5 (4.9%)	x	24.7 (1.4%)
200	153.4(5.5%)	77.4(2.5%)	0.4(0.02%)	1.2 (0.05 %)	115.6(3.9%)	129.8(4.3%)	30(1.1%)	37.4(1.2%)

Supplementary Table 4B

Fecal excretion of chronical treatment

Administration Schedule	Dose (mg/kg)	Formulation	4-HPR	MPR	oxo-4HPR	DH-4HPR
Single admin.	150	NCI-FeR	247.1 (9.2%)	0.4 (0.02%)	103.7 (4.2%)	37.6 (1.41%)
		Bio-nFeR	129.5 (6.1%)	1.6 (0.1%)	108.4 (5.0%)	30.3 (1.4%)
2 wks 1 daily dose	150	NCI-FeR	270 (7.6%)	0.8 (0.0%)	201.1 (6.6%)	62.8 (2.1%)
		Bio-nFeR	97.4 (4.2%)	0.9 (0.0%)	97.5 (4.2%)	30.8 (1.3%)

Supplementary Table 5

Tumor types	Mean concentration ng/ml (±sd)							
	4-HPR		MPR		oxo-4HPR		DH-4HPR	
	Plasma	Tumor	Plasma	Tumor	Plasma	Tumor	Plasma	Tumor
Lung cancer	3446 (66)	2228 (352)	394 (68)	2282 (598)	2142 (374)	2028 (150)	355 (86)	580 (11)
Melanoma	1712 (456)	2126 (684)	190 (32)	707 (476)	1226 (220)	2323(134)	227 (50)	284 (13)
Colon cancer	4987 (2725)	1881 (493)	790 (261)	1090 (503)	3519 (1319)	2514 (189)	478 (257)	329 (45)
